# Functional Properties and Components of *Koenigia alpina* Extract

**DOI:** 10.1111/srt.70102

**Published:** 2024-10-10

**Authors:** Kwan‐Woo Lee, Su‐Hyun Mun, Yeon‐A Kim, Hyo‐Rim Kim, Qinglong Jin, Min‐Ki Lee, Soo Nam Park

**Affiliations:** ^1^ ISTY ON NATURE Suwon‐si Gyeonggi‐do Republic of Korea; ^2^ NBBIO Company Suwon‐si Gyeonggi‐do Republic of Korea; ^3^ Department of Biohealth Engineering College of Science and Convergence Technology Seoul Women's University Nowon‐gu Seoul Republic of Korea

**Keywords:** antiaging bioactivity, anti‐inflammatory, antioxidant, botanical, *Koeniga alpina*, whitening

## Abstract

**Background:**

*Koenigia alpina* (All.) T.M.Schust. & Reveal (alpine knotweed) is a perennial herb belonging to the Polygonaceae family. Several studies have examined Polygonaceae species’ potential applications as cosmeceutical materials; however, the potential of *K. alpina* as a cosmeceutical has not yet been studied.

**Materials and Methods:**

Hydrogen peroxide (H_2_O_2_) and lipopolysaccharide were used to induce an inflammatory response in RAW 264.7 cells. 2,2‐Diphenyl‐1‐picrylhydrazyl (DPPH) radicals and H_2_O_2_ were used to evaluate the free‐radical scavenging activity of *K. alpina* extract and its protective effect against reactive oxygen species (ROS)‐induced cell damage. The whitening, antiaging, and cell proliferation/migration effects of the extracts were evaluated via tyrosinase inhibition, collagenase/elastase inhibition, and wound healing assays, respectively. The anti‐inflammatory effect was confirmed by evaluating nitric oxide (NO) production in RAW 264.7 cells. High‐performance liquid chromatography (HPLC), UV, and MS/MS were used to determine the main components of the extract and fractions.

**Results:**

The ethyl acetate (EA) fraction and its aglycone fraction showed very high free‐radical scavenging activities (47.5 and 47.1 µg/mL, respectively). The extract/fractions also showed significant tyrosinase inhibition (IC_50_ = 0.38 mg/mL in EA fraction), collagenase inhibition (IC_50_ = 0.21 mg/mL in EA fraction), and elastase inhibition (IC_50_ = 0.57 mg/mL in aglycone fraction). NO production in lipopolysaccharide‐induced RAW 264.7 cells was inhibited by the extract/fractions. The extract also promoted the closure of scratch wounds in HaCaT cells. The *K. alpina* extract/fractions contained cardamonin, quercetin, and quercitrin.

**Conclusion:**

*K. alpina* extracts/fractions showed antioxidant, antiaging, whitening, and anti‐inflammatory activities, suggesting they may have potential as antiaging cosmeceuticals.

## 1 Introduction

Skin aging can be classified into “intrinsic aging,” a degenerative process resulting from a decline in internal physiological functions, and “extrinsic aging,” caused by external environmental factors such as ultraviolet (UV) radiation and pollution. Skin aging caused by repeated exposure to UV radiation is known as “photoaging.” UV radiation affects keratinocytes and fibroblasts, thereby causing skin dryness, reduced elasticity, abnormal hyperpigmentation disorders, fine and rough wrinkles, and skin sagging [1, 2, 3, 4].

The spectrum of UV can be divided into UVA (315–400 nm), UVB (280–315 nm), and UVC (200–280 nm). UVC is absorbed by the stratospheric ozone layer and does not reach the earth's surface, whereas UVA accounts for up to 95% of the UV radiation that reaches the earth's surface. UVA and UVB exposure induces oxidative stress in human skin, causing DNA damage, upregulation of activator protein 1 (AP‐1) activity, and increased expression of matrix metalloproteinases (MMPs), accelerating photoaging [3, 5–8]. In particular, UVA is a major cause of photoaging because it generates excessive reactive oxygen species (ROS) through photochemical reactions induced by photosensitizers. UV exposure generates ROS such as singlet oxygen (^1^O_2_), superoxide anion radicals (O_2_^•−^), hydrogen peroxide (H_2_O_2_), and hydroxyl radicals (•OH). Excessive ROS overwhelm the skin's antioxidant defenses, leading to oxidative modification of lipids, proteins, and DNA in skin cells, ultimately damaging the structure of skin tissue [9, 10, 11, 12].

ROS induced by UV radiation also activates mitogen‐activated protein kinases, such as extracellular signal‐regulated kinase (ERK), Jun‐N‐terminal kinase, and p38. This activation, in turn, stimulates the transcription of activator protein 1 (AP‐1), which comprises Jun and Fos family proteins (such as c‐Jun and c‐Fos), leading to the upregulation of MMPs, such as MMP‐1 and MMP‐3 [3, 7, 12, 13]. MMPs induce the degradation of extracellular matrix (ECM) proteins, such as collagen and elastin, accelerating skin photoaging. Therefore, to prevent UV‐induced skin photoaging, the key objective is to inhibit ROS production and MMP synthesis or to suppress the activity of MMPs such as collagenase and elastase.

Melanin is synthesized to protect the skin from UV radiation. However, repeated UV exposure can excessively produce and accumulate melanin. Abnormal melanin production is associated with various skin conditions, including melasma, freckles, age spots, and hyperpigmentation disorders [14, 15, 16]. When the skin is exposed to UV radiation, keratinocytes release α‐melanocyte‐stimulating hormone (α‐MSH) and other signaling molecules. α‐MSH binds to receptors on melanocytes, triggering the cAMP–PKA–CREB–MITF signaling pathway, leading to the expression of melanin‐synthesizing enzymes, such as tyrosinase. Melanin production in the skin begins in the melanosomes of melanocytes through the action of these enzymes. Melanin is ultimately classified into two types: eumelanin and pheomelanin. Eumelanin and pheomelanin are two distinct types of melanin that determine skin, hair, and eye pigmentation. Eumelanin is responsible for the appearance of black and brown colors and provides significant protection against UV radiation, helping to shield the skin from UV damage. It is predominant in darker hair and skin tones. On the other hand, pheomelanin gives rise to yellow and red hues and is found in lighter skin tones and red hair. It offers less UV protection than eumelanin. The balance between these two types of melanin, which is influenced by genetic factors, determines an individual's overall pigmentation and susceptibility to UV radiation. The synthesis of melanin starts with the hydroxylation of L‐tyrosine to L‐dihydroxyphenylalanine (L‐DOPA) and its subsequent oxidation to L‐dopaquinone (DQ). This reaction is catalyzed by tyrosinase (the main rate‐limiting enzyme in the synthesis of eumelanin and pheomelanin), the first of three important enzymes in the melanogenic pathway. After DQ is formed, pheomelanin synthesis occurs when DQ and cysteine react to form cysteine‐DOPA, which is then converted to quinoline and finally polymerized into pheomelanin. For eumelanin synthesis, DQ undergoes cyclization to form dopachrome, which can spontaneously convert into 5,6‐dihydroxyindole or be enzymatically converted into 5,6‐dihydroxyindole‐2‐carboxylic acid by another key enzyme, tyrosinase‐related protein 2 (Trp2). The final conversion to eumelanin occurs through the spontaneous polymerization of 5,6‐dihydroxyindole or the polymerization of 5,6‐dihydroxyindole‐2‐carboxylic acid, mediated by a third enzyme, tyrosinase‐related protein 1 (Trp1) [17, 18, 19].

Arbutin is widely used as a functional whitening agent in cosmetics. However, this compound is a synthetic raw material, and the whitening effect is not substantial. Meanwhile, the demand for plant‐derived natural compounds continues to increase owing to nature‐oriented consumer awareness [14, 20, 21]. Therefore, herein, the inhibitory activity of tyrosinase, the first and most important enzyme in melanin production, was measured in *Koenigia alpina* extract, its ethyl acetate (EA) fraction, and the aglycone fraction, and its potential as a whitening material in cosmetics was examined.

Inflammation is a complex process mediated by the activation of various immune cells. Macrophages play a central role in various immunopathological phenomena that occur during inflammation by overproducing inflammatory mediators and pro‐inflammatory cytokines, such as interleukin (IL)‐1β, IL‐6, tumor necrosis factor (TNF)‐α, nitric oxide (NO) (synthesized by inducible NO synthase [iNOS]), and prostaglandin (PG) E2 (synthesized by cyclooxygenase‐2, COX‐2). In mouse macrophage RAW 264.7 cells, the production of NO, an inflammatory mediator, can be induced by lipopolysaccharides (LPS). Therefore, this cell system provides a model for screening and evaluating potential inhibitors of the inflammatory response [22, 23, 24]. Herein, we measured the effects of *K. alpina* extract, EA fraction, and aglycone fraction on the NO production induced by LPS in RAW 264.7 cells and investigated whether they could have anti‐inflammatory effects.

*K. alpina* (All.) T.M.Schust. & Reveal, commonly known as alpine knotweed, is a perennial herb belonging to the Polygonaceae family. Alpine knotweed is widely distributed in temperate and subarctic regions of the Northern Hemisphere, including Korea and China, and commonly grows in mountainous areas. It is native to Europe and temperate Asia. In Korea, three species of *Koenigia* are known to occur naturally: *K. alpina* (All.) T.M. Schust. & Reveal, *K. limosa* (Kom.) T.M.Schust. & Reveal, and *K. divaricata* (L.) T.M. Schust. & Reveal. *K. alpina* (All.) T.M.Schust. & Reveal was used in this study.

Several previous studies have been conducted on several species of the Polygonaceae family to examine their potential application as functional materials for cosmetics [25, 26, 27, 28, 29, 30, 31, 32, 33, 34, 35, 36]. The Polygonaceae plant species studied were *Persicaria perfoliata* L. H. Gross, *Persicaria hydropiper* L. Delarbre, *Polygonum aviculare* L., and *Rumex acetosa* L. (*R. acetosa*). In previous studies, 50% ethanol (EtOH) extract, an EA fraction, and an aglycone fraction as active fractions were prepared from the plants of the Polygonaceae family, and their efficacies were evaluated. In addition, a single compound was isolated from the active fractions, and its efficacy was evaluated. The antioxidant efficacy (DPPH‐free radical scavenging activity and ROS scavenging activity of luminol‐Fe^3+^‐EDTA/H_2_O_2_ system; total antioxidant activity), protective effect on human cells against ROS (^1^O_2_) generated by a photosensitization reaction, antibacterial activity, and protective effect against damage to HaCaT cells owing to ROS (H_2_O_2_ or ^1^O_2_) treatment were also measured. The results showed that the extracts, fractions, and components from Polygonaceae had excellent antioxidant and cell protection effects. Elastic liposomes or CPP‐liposome‐containing extracts or active ingredients from the Polygonaceae family were prepared, and the in vitro skin penetration abilities of these extracts and ingredients were studied. For each formulated liposome, the particle size, zeta potential, encapsulation efficiency, deformability, and skin penetration ability were evaluated. In particular, the cell‐penetrating peptide‐conjugated liposomes containing *P. aviculare* extract considerably improved the skin penetration ability of active ingredients and therefore showed excellent in vivo anti‐wrinkle and depigmentation efficacy [34, 35]. As a result of the aforementioned studies, *P. aviculare* extract and *P. hydropiper* extract have been listed in the Cosmetic Ingredient Dictionary by the Korea Cosmetic Association and are currently used as cosmetic ingredients. The experimental results suggest that extracts and components from plants in the Polygonaceae family not only possess strong antioxidant effects but also show substantial potential as antiaging or whitening functional ingredients in cosmetics, especially when used in conjunction with formulations such as liposomes.

Noting the various skin benefits of extracts, fractions, and components from plants in the Polygonaceae family and their potential applications in cosmetics, we conducted a preliminary study on the 70% EtOH extract, EA fraction, and aglycone fraction of *K. alpina*, a species that has not been previously researched. We evaluated their antioxidant, tyrosinase inhibition, collagenase inhibition, elastase inhibition, cell‐protective, wound‐healing, and anti‐inflammatory (inhibition of NO production) activities, and conducted the component analysis. The objective was to provide foundational data to assess the potential applicability of these substances in cosmetics.

## 2 Materials and Methods

### 2.1 Reagents

2,2‐Diphenyl‐1‐picrylhydrazyl (DPPH), tyrosinase, collagenase, elastase, thiazolyl blue tetrazolium bromide (MTT), sodium nitrite (NaNO_2_), lipopolysaccharide, and Griess reagent were purchased from Merck Korea (Korea). H_2_O_2_ solution was purchased from Green Pharmaceutical Co., Ltd. (Korea). EtOH, EA, and dimethyl sulfoxide solvents were purchased from Daejung Chemicals & Metals Co., Ltd. (Korea). For cell culture, Dulbecco's modified Eagle's medium (DMEM), penicillin‐streptomycin, and trypsin were purchased from Gibco (California, USA). Fetal bovine serum (FBS) was purchased from Hyclone (USA).

### 2.2 Cell Culture

The RAW 264.7 and HaCaT cells used in this study were procured from the Korean Cell Line Bank (Seoul, Korea). Cells were cultured in DMEM (Gibco, California, USA) supplemented with 10% FBS (v/v) and 1% penicillin‐streptomycin in an atmosphere with 5% CO_2_ and 95% relative humidity at 37°C.

### 2.3 Extraction of *K*. *alpina* Flavonoid Fractions

The dried *K*. *alpina* used in this study was obtained from Shaanxi Joryherb Bio‐technology Co., Ltd. (China). It was ultrasonically extracted in 70% EtOH for 3 h at room temperature. Filtered extracts were then concentrated using a rotary evaporator and subsequently dried in a freeze dryer (DAEHAN Scientific Co., Ltd., Korea). Concentrated extracts were fractionated into soluble fractions using EA solvent. The concentrated extract (70% EtOH extract) was extracted three times using EA and water (1:1) to obtain an EA fraction. Then, the water was extracted using Na_2_SO_4_, and the fraction was concentrated under reduced pressure to obtain an EA fraction. A portion of the EA fraction (containing flavonoid glycosides) was subjected to acid hydrolysis to remove the sugars to obtain an aglycone fraction. To achieve this, we added 9 mL of distilled water, 10 mL of acetone, and 1 mL of H_2_SO_4_ (in that order) to 0.2 g of the EA fraction, which was then refluxed and cooled in a water bath for 4 h. The refluxed solution was neutralized with 5% KOH‐MeOH solution, fractionated using EA, and distilled under reduced pressure to prepare the aglycone fraction [37]. Yields of the 70% EtOH extract and EA and aglycone fractions are shown in Table 1. Each fraction was used to evaluate various activities.

**TABLE 1:** Yields of the 70% EtOH extract and its fractions from *K*. *alpina*.

Extract/fraction	Weight (g)	Yield (% w/w)^a^
70% EtOH extract	4.44	4.44
Ethyl acetate fraction	0.95	0.95
Aglycone fraction	0.40	0.40

Abbreviation: EtOH, ethanol.

^a^ Yield (%) was calculated as the extract/fraction dry weight from 100 g of plant material.

### 2.4 Free‐Radical Scavenging Activity

To evaluate the free‐radical scavenging activity of *K*. *alpina* extract, various concentrations of the extract and *K*. *alpina* fractions (0.1 mL of each) were mixed with an EtOH and methanol solution (0.1 mL) containing 0.2 mM DPPH. Scavenged DPPH radicals were measured via absorbance at 517 nm using a UV spectrophotometer. An equal amount of DPPH and methanol served as the standard and the blank, respectively, whereas α‐tocopherol served as the positive control. Scavenging activity was calculated using the following formula:

$$\mathrm{Scavenging}\left(\%\right)=\left(A_{\mathrm{control}}-A_{\mathrm{sample}}\right)/A_{\mathrm{control}}\times 100\%,$$

where *A*_sample_ and *A*_control_ are the absorbances of the test sample and control, respectively [38, 39].

### 2.5 Tyrosinase Inhibition Activity

Tyrosinase, an oxidoreductase involved in melanin biosynthesis, catalyzes the conversion of tyrosine to 3,4‐DOPA and DOPA quinone. By inhibiting the activity of tyrosinase, a key enzyme in melanin production, it is possible to induce a whitening effect by limiting melanin synthesis [40, 41]. Moreover, in developing whitening raw materials, selective inhibition of tyrosinase is crucial as it reduces hyperpigmentation without toxicity to healthy melanocytes [41]. In the present study, tyrosinase was subjected to an inhibition assay using 3,4‐dihydroxy‐1‐phenylalanine (L‐DOPA) as the substrate. The procedure involved mixing various concentrations (100, 200, 500, 1000, and 2000 µg/mL) of EtOH extract, EA fractionation, and aglycone fractionation (40 µL) of *K*. *alpina* with 67 mM sodium phosphate buffer (pH 6.8), followed by the addition of tyrosinase enzyme (40 µL), which was then activated for 10 min in the shade at room temperature. Subsequently, L‐DOPA (40 µL) was added and activated for 3 min under the same conditions. Absorbance was then measured at 475 nm. Inhibition of enzymatic activity was calculated as the half‐maximal inhibitory concentration (IC_50_) by considering the sample without added tyrosinase as the blank.

### 2.6 Collagenase and Elastase Inhibition Activity

Inhibition of collagenases, enzymes that break peptide bonds in collagen, was assayed using a modified ninhydrin assay [42]. Specifically, a solution was prepared by mixing EtOH extract, EA fractionation, and aglycone fractionation (0.01 g) from *K*. *alpina* and anhydrous EtOH (1 mL), and individual samples were prepared for 1000, 2000, 5000, and 10 000 µg/mL. After adding 30 mg of collagen to each conical tube corresponding to the number of samples to be measured, 3 mL of *N*‐tris[hydroxymethyl]‐methyl‐2‐aminoethanesulfonic acid buffer, prepared using 1 L of distilled water, 12.6 g of *N*‐tris[hydroxymethyl]‐methyl‐2‐aminoethanesulfonic acid, and 0.05 g of calcium chloride, was added to each tube. After adding the sample (1 mL) and collagenase solution (1 mL), the enzyme reaction was activated by stirring for 5 h at 37°C. Following this reaction, the reaction solution (2 mL) was mixed with ninhydrin reaction buffer (1 mL) and boiled for 10 min to obtain a purple solution. After cooling to room temperature, the reaction solution was mixed with an equal amount of n‐propanol, and the centrifuged supernatant (12 000 rpm for 10 min) was measured for absorbance at 600 nm. Epigallocatechin gallate (EGCG), which was used as a positive control, exhibited an IC_50_ value of 0.04 mg/mL.

Elevated elastase activity contributes to skin wrinkle formation by reducing elastic fibers in the skin [43]. The elastase inhibitory assay was conducted according to the method of Azmi et al. [44]. Briefly, a mixture containing 100 µL of 0.2 M Tris‐HCl buffer, 25 µL of 10 mM *N*‐(methoxysuccinyl)‐ala‐ala‐pro‐val‐4‐nitroanilide, 50 µL of various concentrations (0.25, 1.25, 2.5, and 5 mg/mL) of EtOH extract, EA fractionation, aglycone fractionation, and 25 µL of 0.3 unit/mL elastase was incubated for 30 min. Subsequently, absorbance at 410 nm was measured, and the IC_50_ was calculated using the measurement without added enzyme as the blank.

### 2.7 Protective Effect of *K*. *alpina* Against ROS‐Induced Cell Damage

To assess the protective effect of *K*. *alpina* against ROS‐induced cytotoxicity in HaCaT cells, we initially evaluated the cytotoxicity of various *K*. *alpina* concentrations and the ROS H_2_O_2_. Exposure of keratinocytes to certain external stimuli leads to oxidative stress, which in turn generates active oxygen that impairs the elasticity and moisturizing function of keratinocytes, accelerating aging and inducing inflammation and necrosis of skin cells [45, 46]. In our assay, HaCaT cells were seeded at 1 × 10^5^ cells per well in 96‐well plates and treated with *K*. *alpina* extract/fractions (1, 10, 50, and 100 µg/mL) and 10 mM H_2_O_2_ for 24 h. Following treatment, cell viability was determined using the MTT assay.

### 2.8 In Vitro Scratch Wound Closure Assay

Cell migration plays a crucial role in the wound repair process [47]. Therefore, wound healing assays are used to investigate cell migration and wound healing [48]. In the present study, a wound‐healing assay was performed to assess the impact of *K*. *alpina* on cell migration in human keratinocytes, following the method described by Balekar et al. [49]. Briefly, HaCaT cells were seeded into 6‐well plates at a concentration of 2 × 10^6^ cells per well in culture medium and incubated for 24 h at 37°C under a 5% CO_2_ atmosphere. The adherent cell layer was vertically scratched using a 200 µL pipette tip, and cellular debris was removed by washing with phosphate‐buffered saline (1 ×). Fresh medium was added, and the cells were treated with various concentrations of *K*. *alpina*, whereas control cells received only fresh DMEM. To inhibit cell proliferation and assess only cell migration, 10 µg/mL mitomycin C was added to each well. Cell migration was observed using a microscope (BestScope, BS‐7000A, China) at 0, 16, and 24 h after sample treatment.

### 2.9 NO Production in RAW 264.7 Macrophages

The measurement of nitrite concentration serves as a common index of NO production [50]. In lipopolysaccharide‐induced RAW 264.7 cells, NO production was determined using the Griess reaction method [51], which quantifies nitrite levels in aqueous solutions. Cells were seeded at a density of 2 × 10^5^ cells per well in 96‐well plates and incubated for 24 h at 37°C under a 5% CO_2_ atmosphere. Subsequently, the cells were treated with various *K*. *alpina* concentrations (1, 5, 10, 50, 100, and 200 µg/mL) in the presence of lipopolysaccharide (100 ng/mL). The supernatant was mixed with an equal volume of Griess reagent (0.04 g/mL) and incubated at room temperature for 10 min. Finally, the nitrite concentrations were determined from a standard curve established using serial dilutions 0, 6.25, 12.5, 25, 50, and 100 µM of NaNO_2_ and measured at 540 nm using a microplate reader.

### 2.10 Component Analysis of *K*. *alpina* Extract

To identify the flavonoid components of *K*. *alpina* extract, analysis was performed using high‐performance liquid chromatography (HPLC). EA fractions were dissolved in EtOH, and extracts were filtered using a syringe filter (Millipore 0.2 µm) before HPLC analysis. HPLC separation conditions are shown in Table 2. The LC instrument was equipped with a YL HPLC System (YL9100 Plus HPLC System; YL Instrument, Gyeonggi‐do, Korea). MS/MS analysis was performed using Thermo Q‐Exactive (Thermo Scientific, USA) in a negative mode, with a negative ion voltage of 3 kV and an ion transfer tube temperature of 320°C.

**TABLE 2:** HPLC conditions for separation of the ethyl acetate fraction from the *K*. *alpina* extract.

Column	Agilent TC‐C18 (4.6 × 250 mm, 5 µm)		
Detector	365 nm		
Flow rate	1.0 mL/min		
Injection volume	20 µL		
Column temperature	30°C		
Mobile phase conditions for HPLC gradient elution	Time (min)	2% AA in water (%)	0.5% AA in 50% ACN (%)
	0	80	20
	35	40	60
	50	40	60
	55	80	20
	60	80	20

Abbreviations: AA, acetic acid; ACN, acetonitrile.

### 2.11 Statistical Analysis

Statistical analysis was performed on data obtained from experiments repeated in triplicate, and results are presented as means ± standard deviations. Statistical comparisons between groups were conducted using one‐way analysis of variance, followed by Tukey's post‐hoc test for multiple comparisons. *p* < 0.05 was considered statistically significant. All analyses were conducted using Microsoft Excel.

## 3 Results

### 3.1 Antioxidant Activity

**FIGURE 1:**
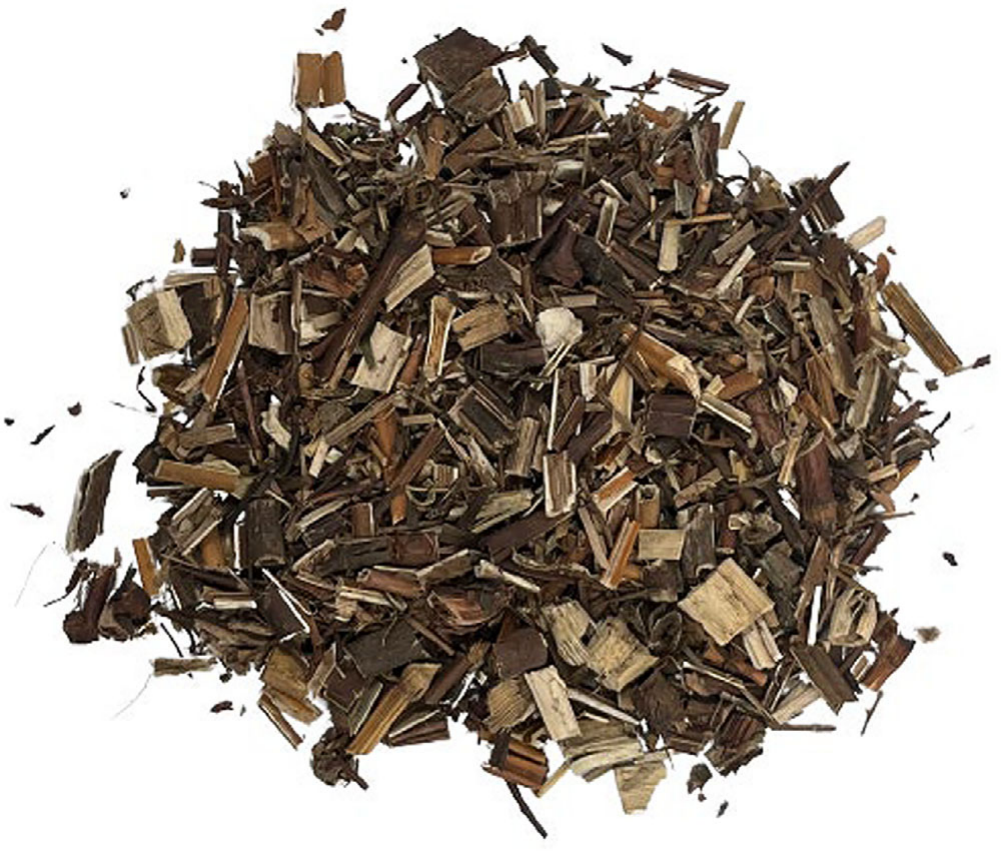
*Koenigia alpina*.

**FIGURE 2:**
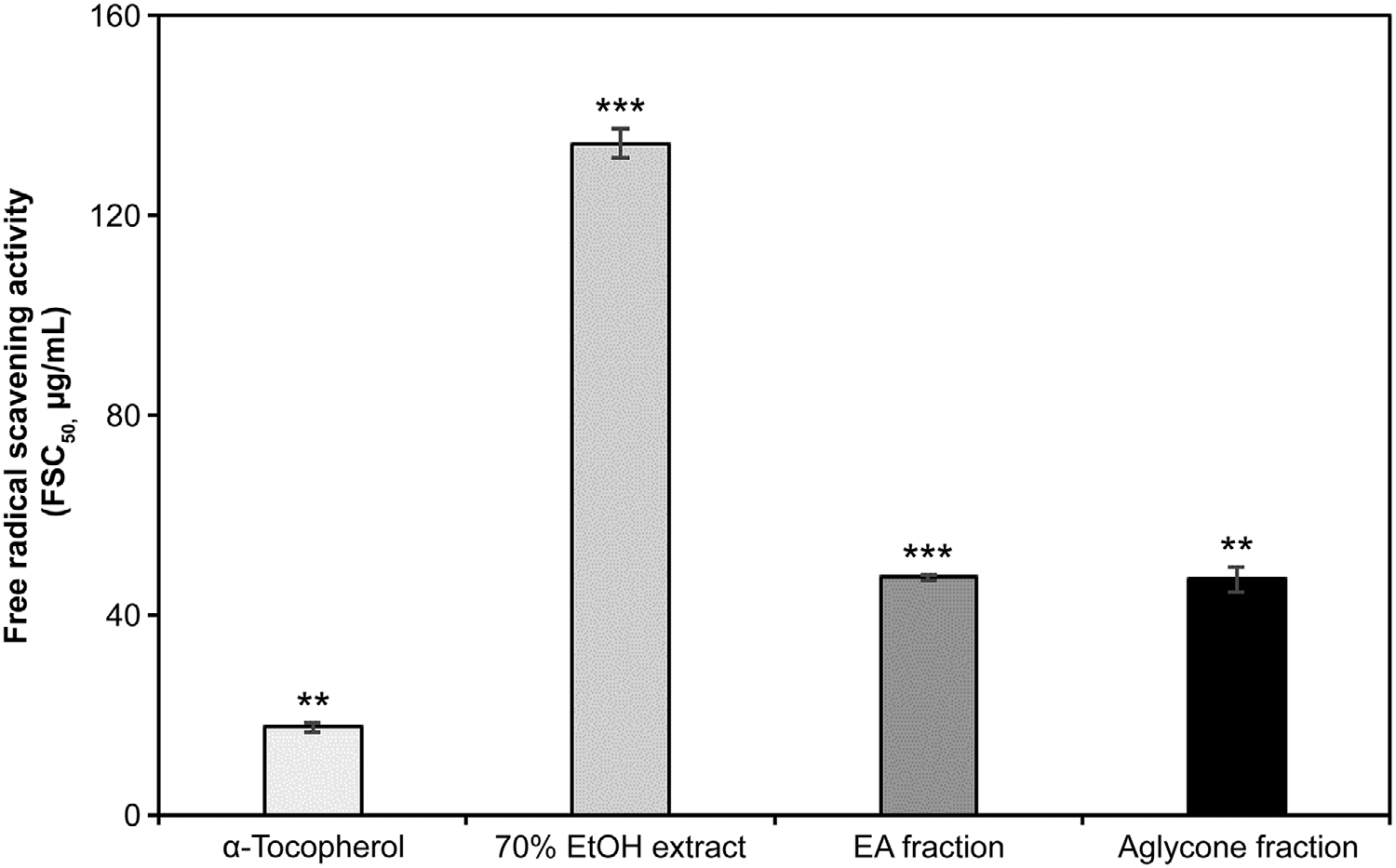
Free‐radical scavenging activities of extract and fractions of *K. alpina*. Each value represents the mean ± SD of three independent experiments. Different letters indicate significant differences (**p < 0.01, ***p < 0.001) based on ANOVA with Tukey's HSD test. EA indicates ethyl acetate; EtOH, ethanol; HSD, honestly significant difference; FSC, The half maximal of free‐radical scavenging concentration.

The fraction extracted from *K. alpina* (Figure 1) exhibited strong scavenging activity compared with α‐tocopherol (vitamin E), a well‐known radical scavenger and antioxidant for lipid bilayers (Figure 2). The free‐radical scavenging activities (FSC_50_) of the *K*. *alpina* extract, EA fraction, and aglycone fraction were 134.2, 47.5, and 47.1 µg/mL, respectively, with the fractions exhibiting the highest scavenging activity. α‐tocopherol, a potent antioxidant, exhibited an FSC_50_ of 17.5 µg/mL.

### 3.2 Tyrosinase Inhibition

**FIGURE 3:**
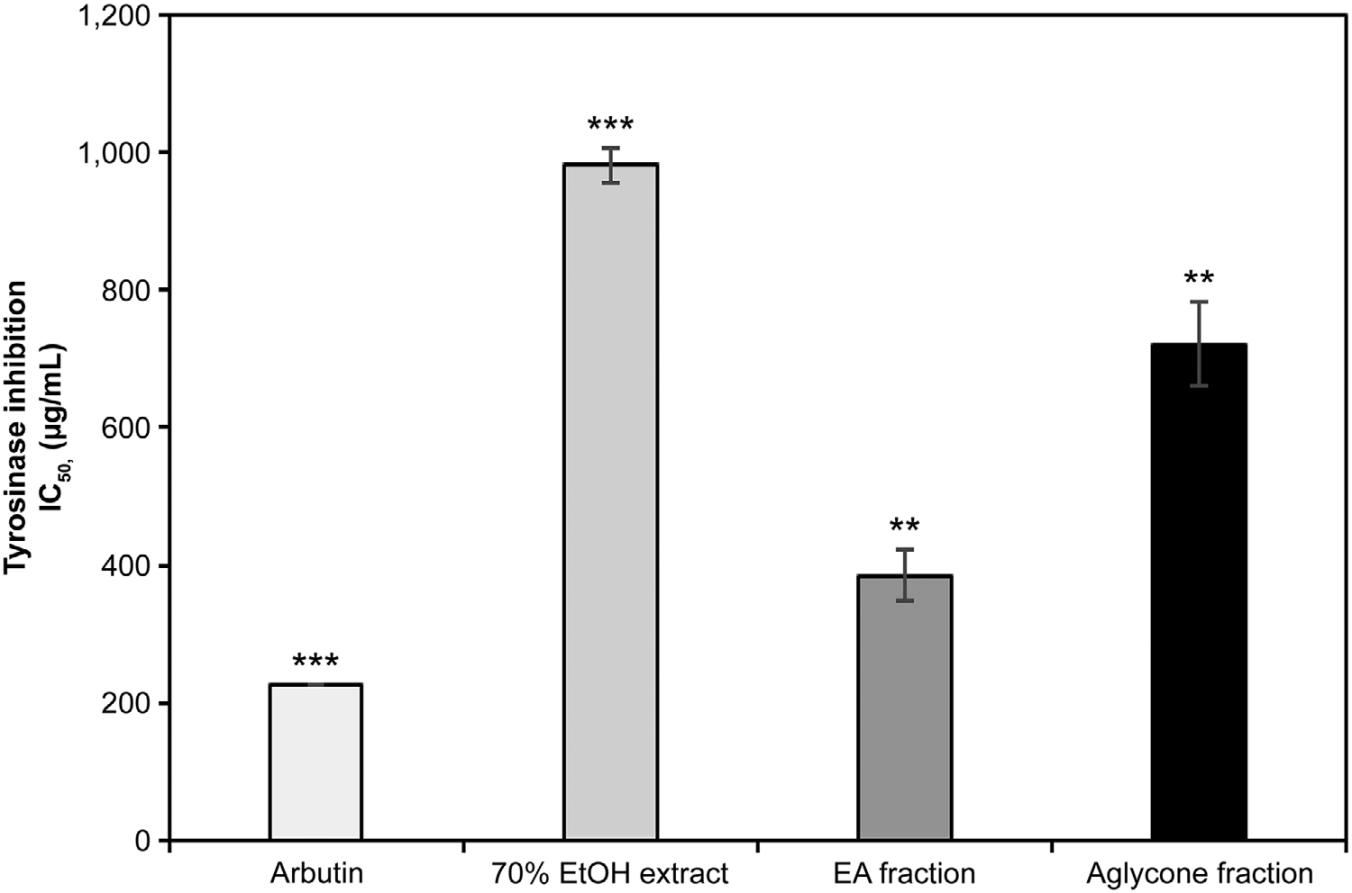
Tyrosinase inhibitory activities of extract and fractions from *K. alpina*. Each value represents the mean ± SD of three independent experiments. Different letters indicate significant differences (**p < 0.01, ***p < 0.001) based on ANOVA with Tukey's HSD test. EA indicates ethyl acetate; EtOH, ethanol; IC, The half maximal Inhibitory.

In the tyrosinase inhibition assay, the 70% EtOH extract, EA fraction, and aglycone fraction of *K*. *alpina* showed IC_50_ values of 0.98, 0.38, and 0.72 mg/mL, respectively (Figure 3); hence, the *K. alpina* EA fraction exhibited the highest inhibitory activity. The functional whitening ingredient, arbutin, which was used as a positive control, showed an IC_50_ of 0.26 mg/mL. Figure 3 shows an IC_50_ of 0.1 mg/mL.

### 3.3 Collagenase and Elastase Inhibition Activity

**FIGURE 4:**
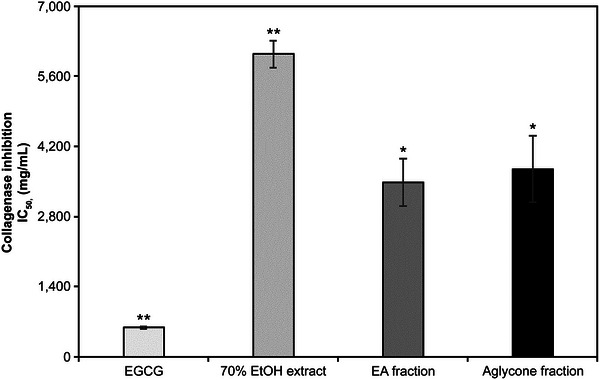
Collagenase inhibitory activities of extract and fractions from *K. alpina*. Each value represents the mean ± SD of three independent experiments. Different letters indicate significant differences (*p < 0.05, **p < 0.01) based on ANOVA with Tukey's HSD test. EA indicates ethyl acetate; EGCG, epigallocatechin gallate; EtOH, ethanol; IC, The half maximal Inhibitory.

**FIGURE 5:**
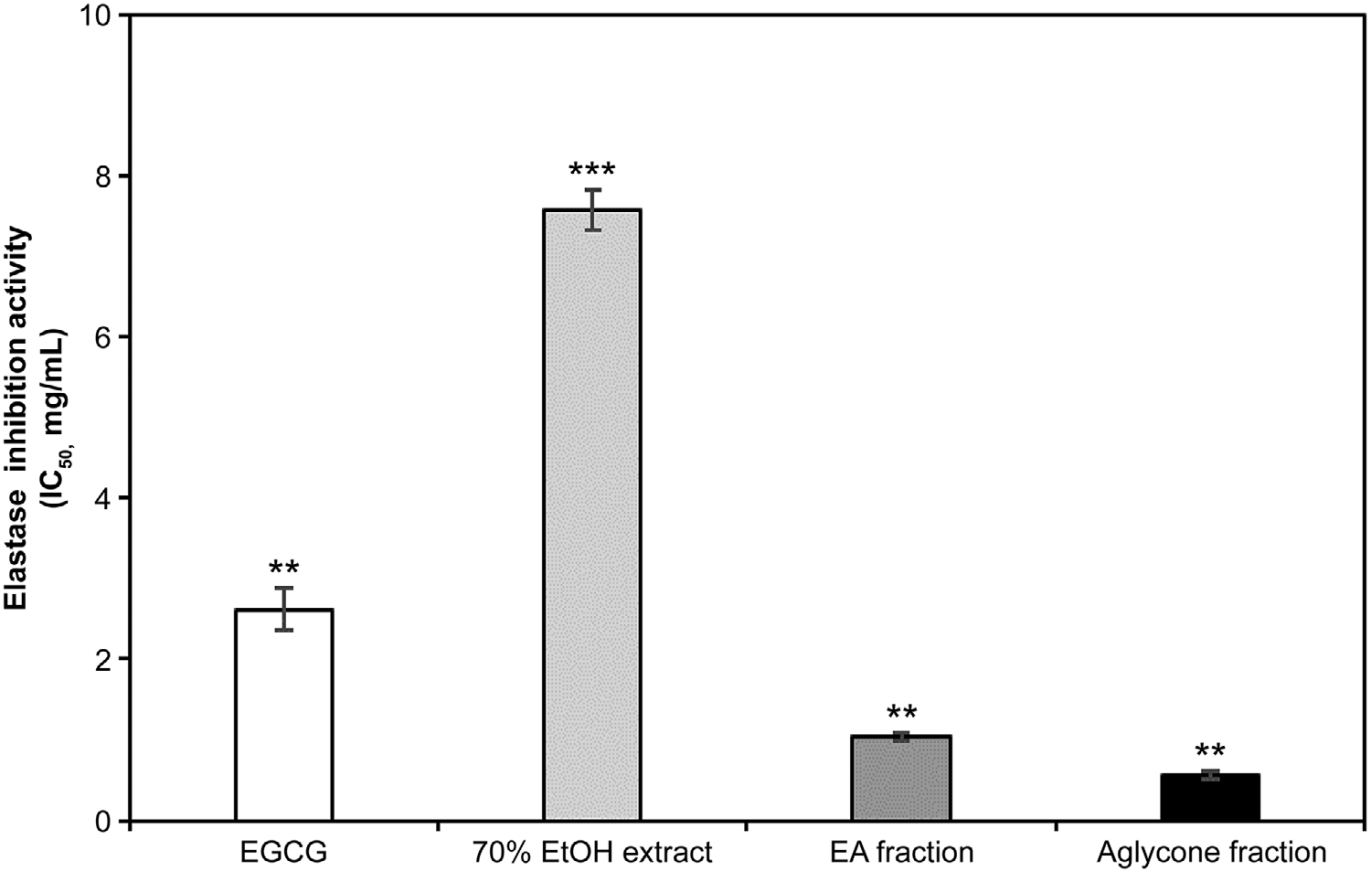
Elastase inhibitory activities of extract and fractions from *K. alpina*. Each value represents the mean ± SD of three independent experiments. Different letters indicate significant differences (**p < 0.01, ***p < 0.001) based on ANOVA with Tukey's HSD test. EA indicates ethyl acetate; EGCG, epigallocatechin gallate; EtOH, ethanol; IC, The half maximal Inhibitory.

In the collagenase inhibition assay, the IC_50_ values for the 70% EtOH extract, EA fraction, and aglycone fraction were 0.39, 0.21, and 0.22 mg/mL, respectively (Figure 4). In the elastase inhibition assay, the IC_50_ values of the 70% EtOH extract, EA fraction, and aglycone fraction were 7.58, 1.03, and 0.57 mg/mL, respectively, with EGCG exhibiting an IC_50_ of 2.62 mg/mL (Figure 5). In both assays, the two *K*. *alpina* fractions showed the highest inhibitory activity.

### 3.4 Effects of *K*. *alpina* Extract on ROS‐Induced Damage in HaCaT Cells

**FIGURE 6:**
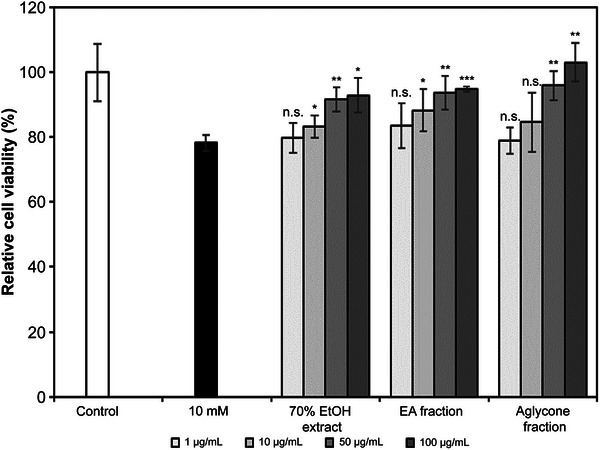
Cellular protective effects of extract and fractions from *K. alpina* on H_2_O_2_‐induced cell damage in HaCaT cells. Cells were treated with 10 mM H_2_O_2_ for 2 h and stained with MTT to compare the number of survival cells between treated and nontreated groups. Each value represents the mean ± SD of three independent experiments. Different letters indicate significant differences (*p < 0.05, **p < 0.01, ***p < 0.001) based on ANOVA with Tukey's HSD test. EA indicates ethyl acetate; EtOH, ethanol; ns, not significant.

Oxidation‐induced cellular damage was restored to normal control levels with 1−100 µg/mL treatments of *K*. *alpina* extract (Figure 6). Thus, *K*. *alpina* extract was capable of protecting HaCaT cells against H_2_O_2_‐induced oxidative stress.

### 3.5 In Vitro Scratch Wound Closure Assay

**FIGURE 7:**
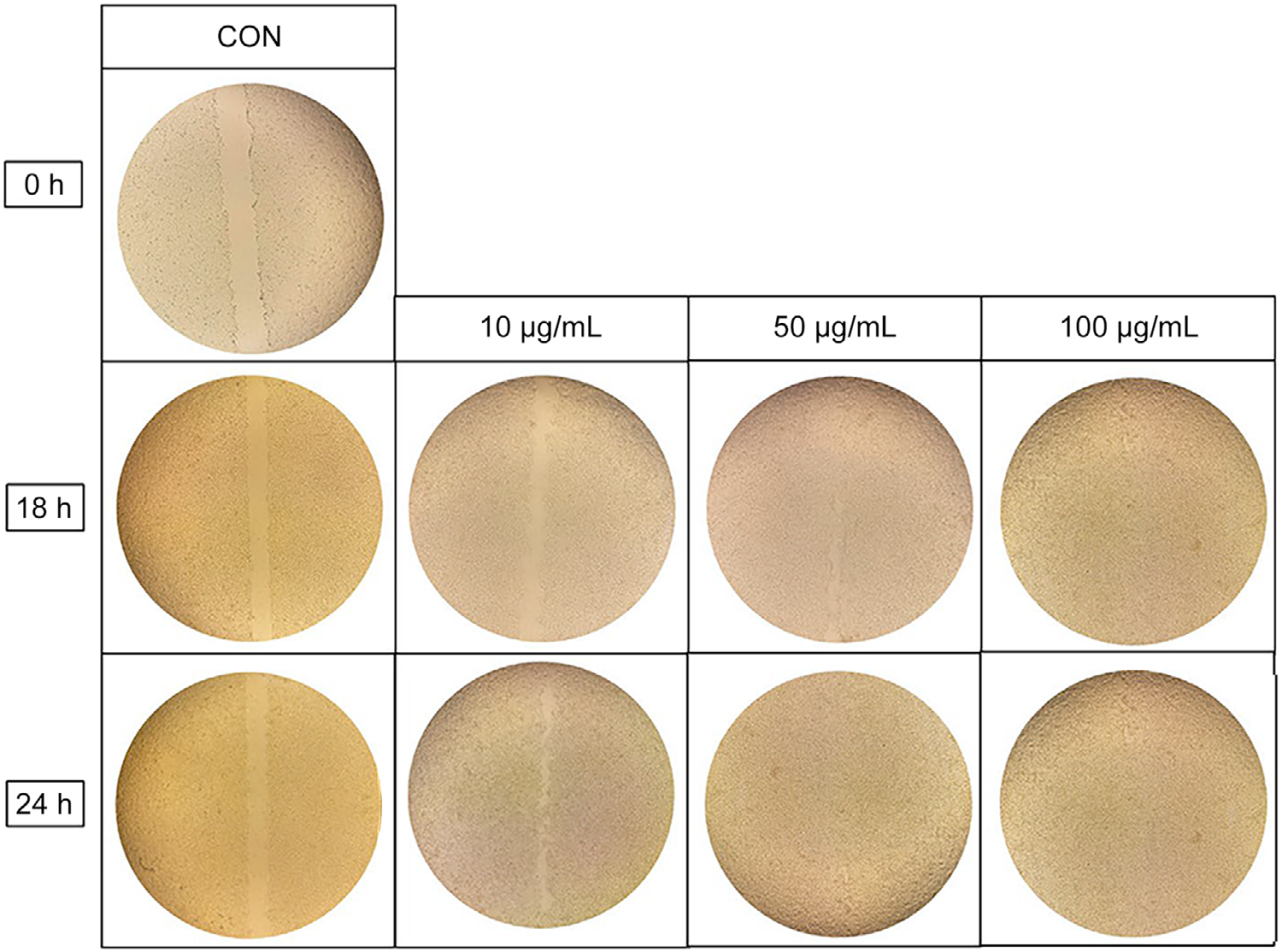
Images from scratch assay of HaCaT cells treated with the extract of *K*. *alpina* compared with untreated control. CON, Negative control.

The cell migration ability of the *K. alpina* extract was assessed by in vitro scratch assay using HaCaT cells (Figure 7). After treating scratched HaCaT cells with *K. alpina* extract at different concentrations (10, 50, and 100 µg/mL), the scratch area was confirmed to be filled at 18 and 24 h. The analysis of the HaCaT cell images showed that the area of the scratch gap decreased in a concentration‐ and time‐dependent manner.

### 3.6 Anti‐Inflammatory Action of *K*. *alpina* Extract on Macrophages

**FIGURE 8:**
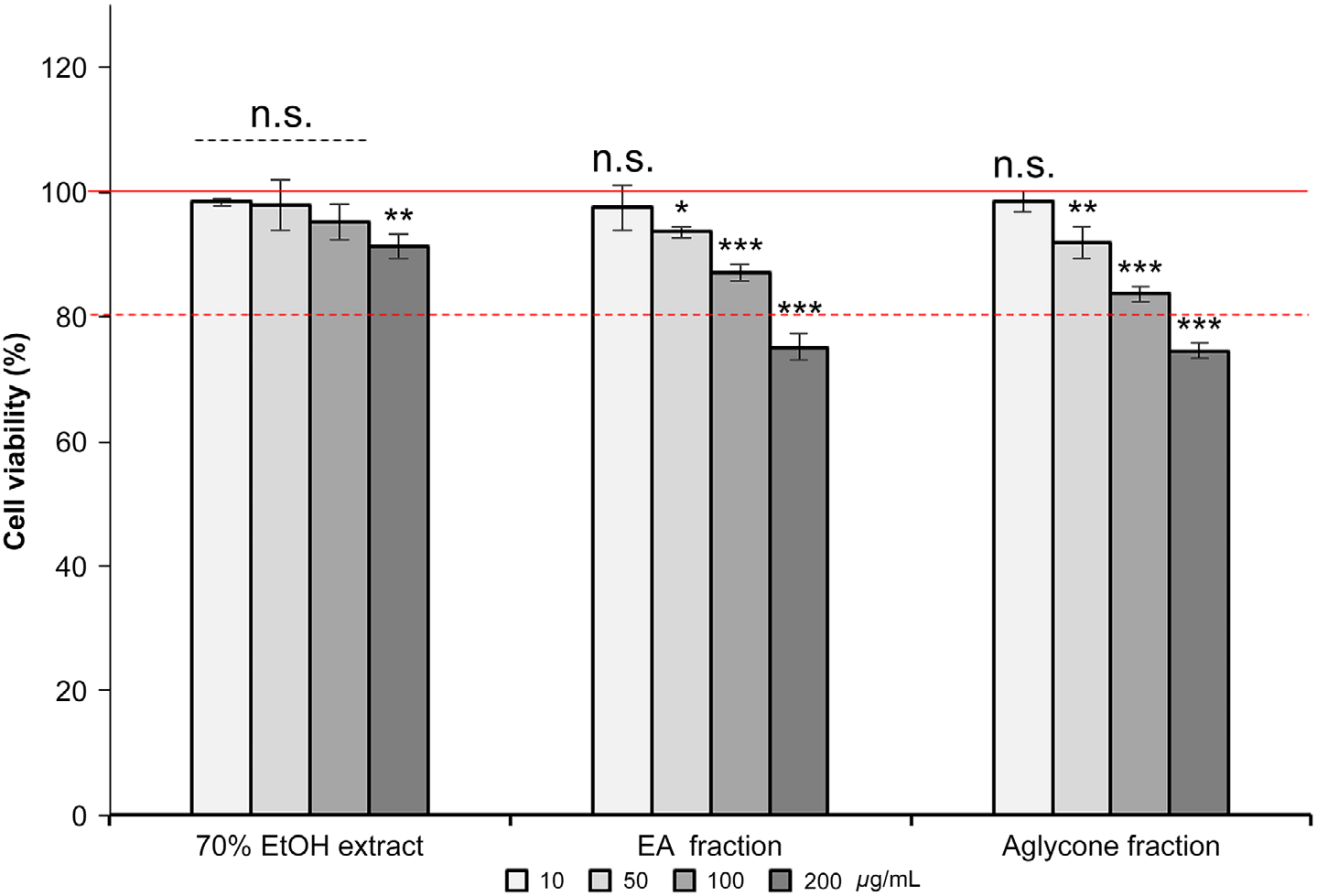
Effects of *K. alpina* extract/fractions on the viability of RAW 264.7 cells. Cells were treated with the indicated concentrations of the 70% EtOH extract and fractions (ethyl acetate and aglycone) for 24 h. Each value represents the mean ± SD of three independent experiments. Different letters indicate significant differences (*p < 0.05, **p < 0.01, ***p < 0.001) based on ANOVA with Tukey's HSD test. EA indicates ethyl acetate; EtOH, ethanol; ns, not significant.

**FIGURE 9:**
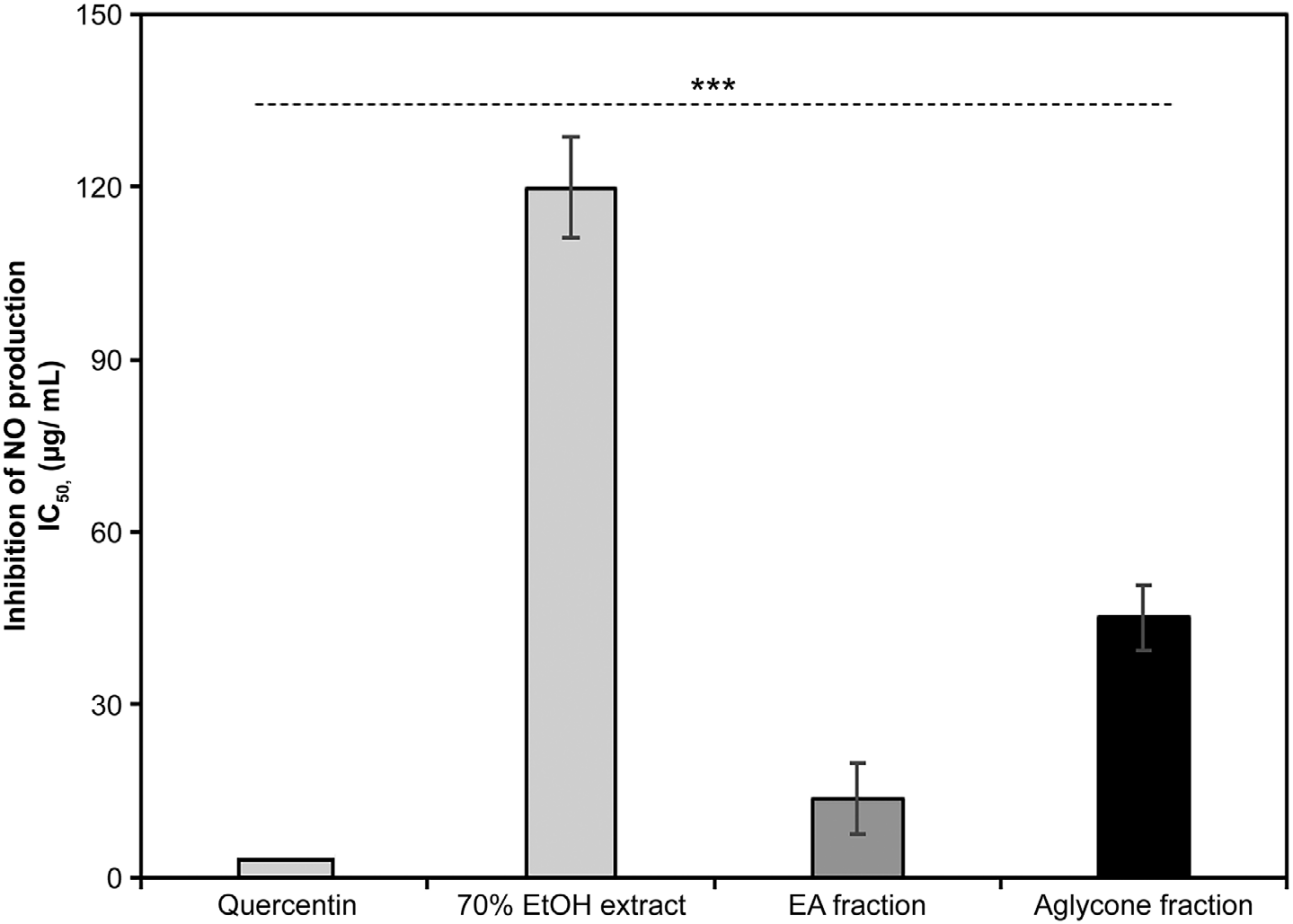
Effects of extract and fractions from *K. alpina* on lipopolysaccharide (LPS)‐induced NO production in RAW 264.7 cells. Each value represents the mean ± SD of three independent experiments. Different letters indicate significant differences (***p < 0.001) based on ANOVA with Tukey's HSD test. EA indicates ethyl acetate; EtOH, ethanol; NO, nitric oxide; IC, The half maximal Inhibitory.

NO plays a pivotal role in inflammation processes; therefore, the NO assay was used to determine the anti‐inflammatory effects of *K*. *alpina* extract by measuring the amount of NO secreted upon macrophage activation. RAW 264.7 cells treated with *K*. *alpina* extract at concentrations of 100 µg/mL exhibited viabilities of 95.3%, 87.0%, and 83.7%, respectively (Figure 8). Moreover, *K*. *alpina* extract reduced lipopolysaccharide‐stimulated NO production in RAW 264.7 macrophages at concentrations that did not affect cell viability. The *K*. *alpina* extract and fractions showed concentration‐dependent activity, with the EA fraction exhibiting the most significant reduction in nitrite levels. IC_50_ values were calculated based on concentrations at which NO levels were reduced by 50%; the IC_50_ values for the 70% EtOH extract, EA fraction, and aglycone fraction were 119.8%, 13.6%, and 45.1%, respectively, with the EA fraction displaying the most pronounced inhibitory activity (Figure 9).

### 3.7 Component Analysis

**FIGURE 10:**
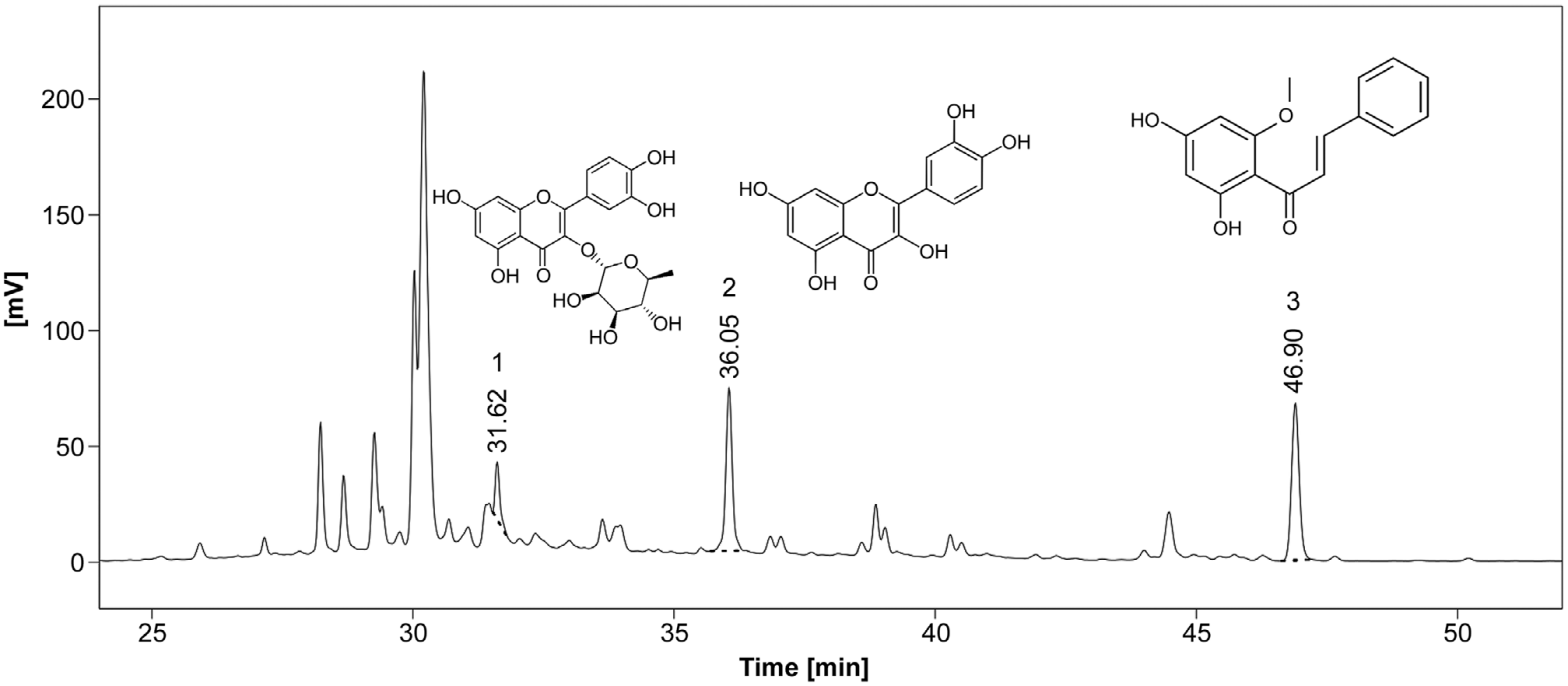
HPLC chromatograms of the ethyl acetate fraction from *K. alpina* extract at a *λ*_max_ of 365 nm.

**FIGURE 11:**
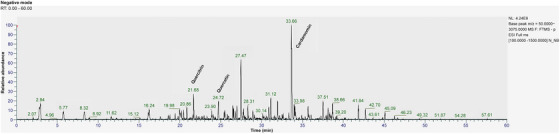
LC‐MS/MS chromatograms of the ethyl acetate fraction from *K. alpina* extract in negative mode.

**FIGURE 12:**
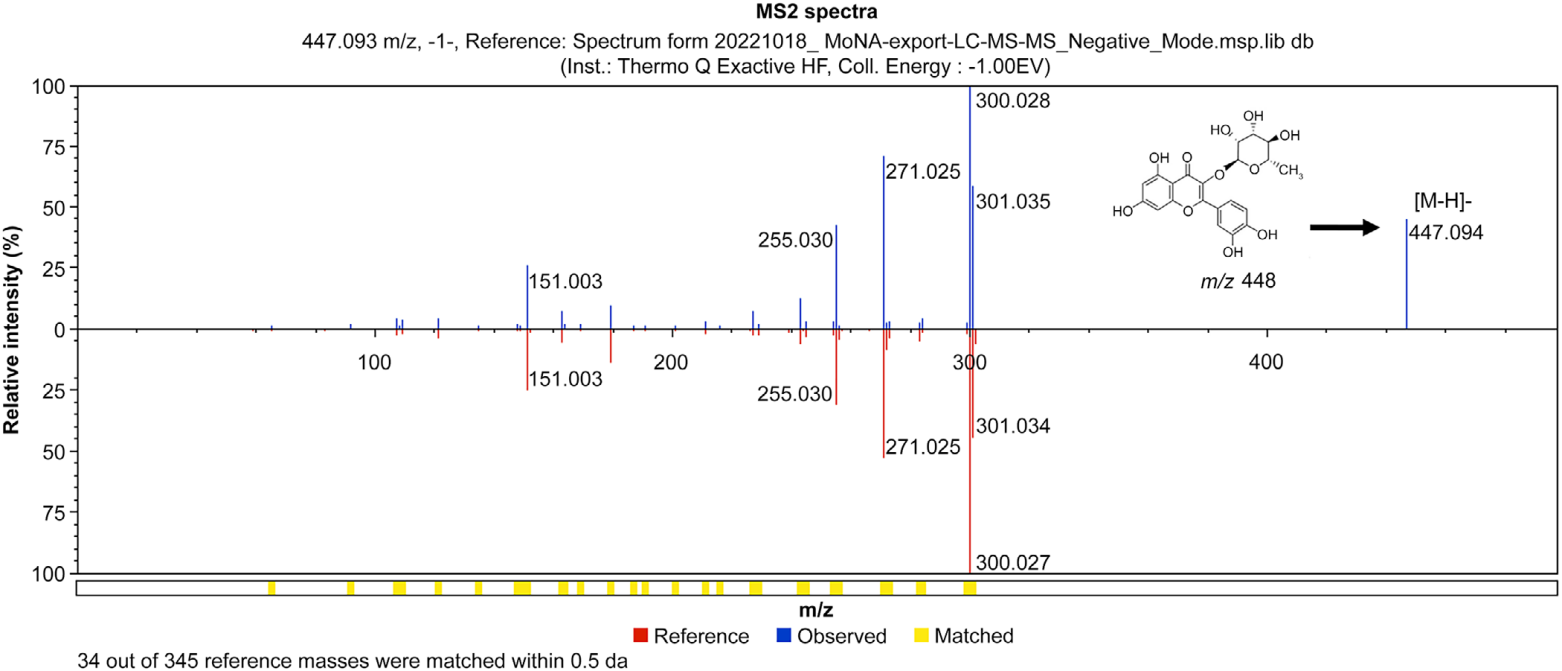
LC‐MS/MS chromatograms of quercitrin in negative mode.

**FIGURE 13:**
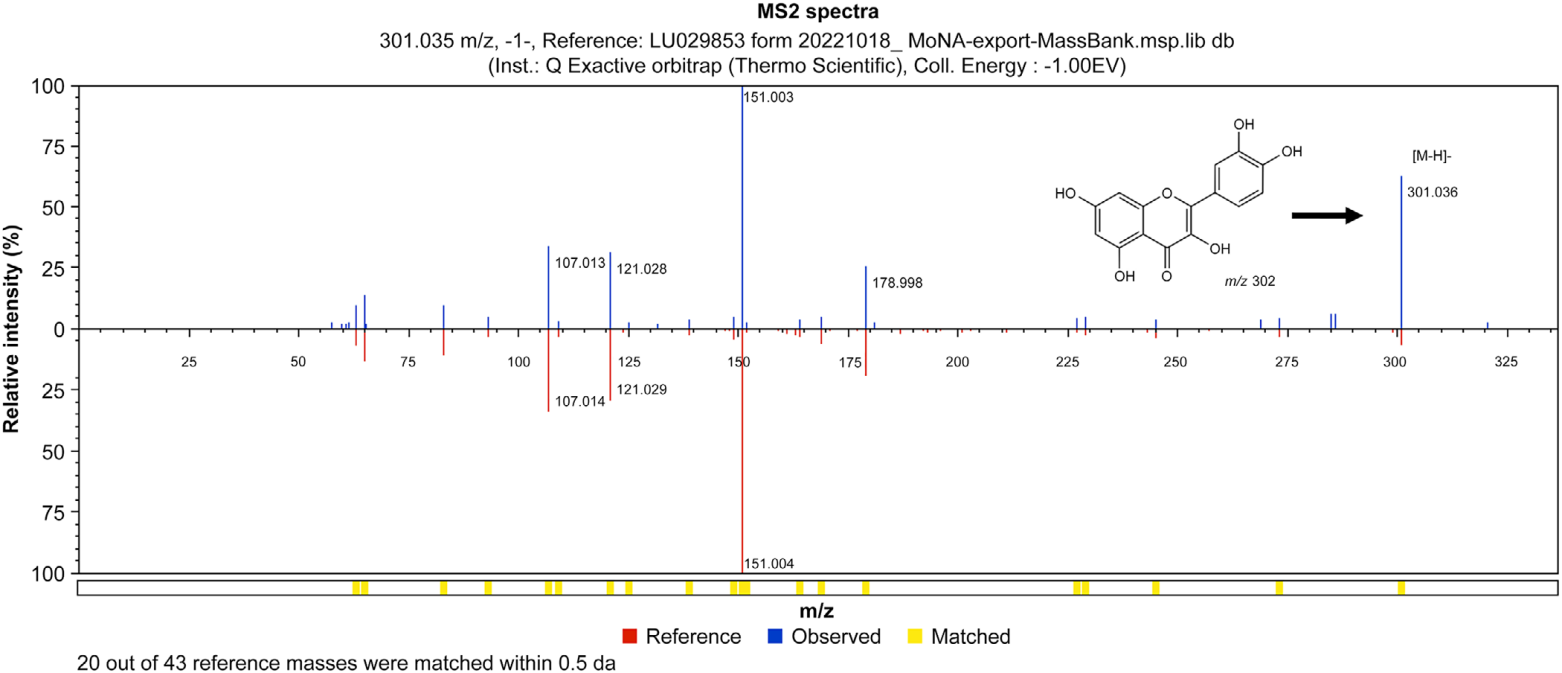
LC‐MS/MS chromatograms of quercetin in negative mode.

**FIGURE 14:**
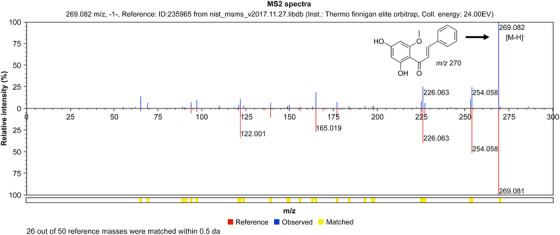
LC‐MS/MS chromatograms of cardamonin in negative mode.

The HPLC chromatogram of the EA fraction of the *K. alpina* extract is shown in Figure 10. The retention time (RT) of the Peak 1 component was 31.62 min, and the UV *λ*_max_ (EtOH) was 253/344 nm; the RT of the Peak 2 component was 36.05 min, and the UV *λ*_max_ (EtOH) was 256/372 nm; the RT of the Peak 3 component was 46.90 min, and the UV *λ*_max_ (EtOH) was 342 nm. Based on the results shown in Figure 10, LC‐MS/MS analysis was conducted. The LC‐MS/MS confirmed that quercitrin, quercetin, and cardamonin were present in the EA fraction of the *K. alpina* extract (Figure 11). The LC‐MS spectrum of HPLC Peak 1 in negative mode showed a molecular ion [M‐H]⁻ at 447 *m*/*z* (Figure 12). The LC‐MS spectrum of the HPLC Peak 2 in negative mode showed a molecular ion [M‐H]⁻ at 301 *m*/*z* (Figure 13). The LC‐MS spectrum of the HPLC Peak 3 in negative mode showed a molecular ion [M‐H]⁻ at 269 *m*/*z* (Figure 14).

The RTs, UV *λ*_max_ (EtOH), and molecular ion [M‐H]⁻ (*m*/*z*) values from the HPLC and LC‐MS spectra, presented in Figures 10, 11, 12, 13, 14, were compared with those of standard compounds. The results of these comparisons are summarized in Table 3 [38, 52–54].

**TABLE 3:** Characteristics and identification of components in the ethyl acetate fraction of *K. alpina* extract.

HPLC peak no.	Retention time (min)	UV *λ*_max_ (EtOH) nm	[M‐H]^−^ (*m*/*z*)	Identified compounds
1	31.62	253, 344	447	Quercitrin^a^
2	36.05	256, 372	301	Quercetin^b^
3	46.90	301	269	Cardamonin^c^

^a^ Quercitrin: 3′,4′,5,7‐tetrahydroxy‐3‐(α‐L‐rhamnopyranosyloxy)flavone.

^b^ Quercetin: 3,3′,4′,5,7‐pentahydroxyflavone.

^c^ Cardamonin: 2′,4′‐dihydroxy‐6′‐methoxychalcone.

### 3.8 Statistical Analysis

Each value presented in the results is the average (mean) plus or minus the standard deviation (SD) derived from three separate and independent experiments. The use of different letters in the data indicates that there are statistically significant differences between the groups. These differences are determined using ANOVA (Analysis of Variance) followed by Tukey's Honestly Significant Difference (HSD) test. Significance levels are categorized as follows: *** denotes a *p* value less than 0.001, ** denotes a *p* value less than 0.01, and * denotes a *p* value less than 0.05. The notation *ns indicates that the difference is not statistically significant.

## 4 Discussion

In this study, we demonstrated that the EtOH extract and its fractions from *K*. *alpina* exhibit significant DPPH radical scavenging, collagenase/elastase/tyrosinase inhibition, anti‐inflammatory, and cellular protective effects. The *K*. *alpina* extract and fractions showed robust antioxidant activity comparable to that of α‐tocopherol, a potent antioxidant. Additionally, they exhibited concentration‐dependent cellular protective effects against ROS‐induced oxidative damage in HaCaT cells. Tyrosinase inhibition, involved in melanin biosynthesis, is crucial for treating hyperpigmentation [55]. The *K*. *alpina* extract and fractions inhibited tyrosinase in a concentration‐dependent manner, with the EA fraction showing inhibition activity as effective as *Glycyrrhiza glabra* extract, a well‐known whitening material. Furthermore, the extract and fractions increased collagenase and elastase inhibitory activity. Collagen and elastin, vital dermal components, maintain skin flexibility and elasticity. These ECM proteins are gradually reduced by degrading enzymes (collagenase and elastase) with age, leading to skin wrinkles [56, 57]. The *K*. *alpina* fractions showed superior elastase inhibition activity compared with the positive control EGCG.

Cell senescence, driven by inflammatory mechanisms, can be delayed by natural polyphenols [58, 59]. In the present study, both *K*. *alpina* fractions showed significant inhibition of NO production, with the EA fraction displaying the greatest inhibitory activity (an IC_50_ of 13.6 µg/mL, approximately 9.1‐fold lower than that of the 70% EtOH fraction), comparable to that of quercetin. These results indicate that the *K*. *alpina* extract and fractions can inhibit active oxygen, skin aging enzyme activity, and inflammageing.

The significant activities of the *K*. *alpina* extract observed in this study can be attributed to its antioxidant, antiaging, whitening, cell protection, and anti‐inflammatory effects. External stimuli, including UV radiation, accelerate the skin's natural aging process, characterized by wrinkles and atypical pigmentation [60]. Our findings provide evidence of *K*. *alpina* extract's efficacy as a possible skin treatment and will contribute to further investigation and development of this candidate substance for application as a natural cosmetic material effective against skin aging and diseases. Despite these advantages, this study has a limitation. In this study, we conducted a component analysis of ingredients derived from *K*. *alpina*; however, we were unable to evaluate the efficacy of each identified component. In this study, we investigated the efficacy of 70% EtOH extracts, EA fraction, and aglycone fraction, to precisely identify the active components of *K. alpina* through further research in the future.

## 5 Conclusion

Herein, the 70% EtOH extract, an EA fraction, and an aglycone fraction were prepared from *K. alpina*, and the extracts and fractions were tested for DPPH‐free radical scavenging, collagenase and elastase inhibitory, tyrosinase inhibitory, anti‐inflammatory, and cytoprotective activities; the extract/fractions were shown to be effective.

The 70% EtOH extract, EA fraction, and aglycone fraction of *K. alpina* showed very high antioxidant activity. In particular, the free‐radical scavenging activities of the EA fraction and aglycone fraction of *K. alpina* were 47.5 and 47.1 µg/mL, respectively. In addition, the *K. alpina* extracts and fractions showed a concentration‐dependent cytoprotective effect against ROS (H_2_O_2_)‐induced cell damage in HaCaT cells. Inhibition of tyrosinase, a key enzyme involved in melanin biosynthesis, can improve hyperpigmentation. The *K. alpina* extracts and fractions also inhibited tyrosinase in a concentration‐dependent manner. Among them, the EA fraction showed excellent tyrosinase inhibitory activity (IC_50_ = 0.38 mg/mL), which was comparable with that of oil‐soluble licorice extract, a well‐known whitening ingredient. The *K. alpina* extracts and fractions also showed good inhibitory activities against collagenase and elastase, which are important for elasticity, were also shown to be good in the dermal layer of the skin. These are important dermal ECM components that maintain skin flexibility and elasticity; in particular, the EA fraction of *K. alpina* showed high collagenase inhibitory activity (IC_50_ = 0.21 mg/mL), and the aglycone fraction showed substantial elastase inhibitory activity (IC_50_ = 0.57 mg/mL). The *K. alpina* fractions showed superior inhibitory activity against elastase compared with EGCG, the main component of green tea. When skin is exposed to UV rays, it can cause photoaging of the skin and skin hyperpigmentation, such as age spots and freckles, through the production of ROS. In addition, skin aging can progress through inflammatory mechanisms, and natural polyphenols or triterpenoids can delay cellular aging. Both fractions of *K. alpina* considerably inhibited NO production, and the EA fraction showed the greatest inhibitory activity (IC_50_ = 13.6 µg/mL). Collectively, these results indicate that the 70% EtOH extract, EA fraction, and aglycone fraction of *K. alpina* has the ability to suppress ROS, the activity of extracellular matrix decomposition enzymes, and inflammation.

The main activities of *K. alpina* extracts and fractions in this study can be explained by their antioxidant, anti‐aging, whitening, cytoprotective, and anti‐inflammatory effects. These results suggest that the 70% EtOH extract, EA fraction, and aglycone fraction of *K. alpina* have the potential to be applied as cosmetic functional materials that are effective against various skin diseases, such as skin aging/pigmentation and inflammation caused by ROS.

## Ethical Statement

This study adheres to the ethical principles and guidelines of the Society of Cosmetic Scientists of Korea. Ethical approval was obtained from NBBIO Company.

## Conflicts of Interest

The authors declare no conflicts of interest.

## Data Availability

The datasets used and/or analyzed during this study are available from the corresponding author on a reasonable request.
